# The dynamics of introgression across an avian radiation

**DOI:** 10.1002/evl3.256

**Published:** 2021-09-28

**Authors:** Sonal Singhal, Graham E. Derryberry, Gustavo A. Bravo, Elizabeth P. Derryberry, Robb T. Brumfield, Michael G. Harvey

**Affiliations:** ^1^ Department of Biology California State University, Dominguez Hills Carson California 90747; ^2^ Department of Ecology and Evolutionary Biology University of Tennessee Knoxville Tennessee 37996; ^3^ Department of Organismic and Evolutionary Biology Harvard University Cambridge Massachusetts 02138; ^4^ Museum of Comparative Zoology Harvard University Cambridge Massachusetts 02138; ^5^ Museum of Natural Science Louisiana State University Baton Rouge Louisiana 70803; ^6^ Department of Biological Sciences Louisiana State University Baton Rouge Louisiana 70803; ^7^ Department of Biological Sciences The University of Texas at El Paso El Paso Texas 79968; ^8^ Biodiversity Collections The University of Texas at El Paso El Paso Texas 79968

**Keywords:** Diversification, gene tree discordance, hybridization, incomplete lineage sorting, introgression, Neotropics, ornithology, phylogenomics, speciation, suboscines

## Abstract

Hybridization and resulting introgression can play both a destructive and a creative role in the evolution of diversity. Thus, characterizing when and where introgression is most likely to occur can help us understand the causes of diversification dynamics. Here, we examine the prevalence of and variation in introgression using phylogenomic data from a large (1300+ species), geographically widespread avian group, the suboscine birds. We first examine patterns of gene tree discordance across the geographic distribution of the entire clade. We then evaluate the signal of introgression in a subset of 206 species triads using Patterson's *D*‐statistic and test for associations between introgression signal and evolutionary, geographic, and environmental variables. We find that gene tree discordance varies across lineages and geographic regions. The signal of introgression is highest in cases where species occur in close geographic proximity and in regions with more dynamic climates since the Pleistocene. Our results highlight the potential of phylogenomic datasets for examining broad patterns of hybridization and suggest that the degree of introgression between diverging lineages might be predictable based on the setting in which they occur.

**Impact Summary**

Hybridization and the genetic introgression between lineages that may result are a fundamental part of the speciation process. Hybridization can prevent speciation by homogenizing gene pools or facilitate speciation by producing new combinations of genetic variants pulled from different lineages. Thus, understanding how and why hybridization varies across geographic regions or lineages may reveal why speciation is more frequent or occurs differently in particular situations. Here, we provide one of the first investigations of speciation dynamics across lineages and regions using phylogenomic data from a large, widespread group of birds, the suboscines. We show that some regions and lineages have higher rates of two metrics related to introgression—gene tree discordance and introgression signal—and that the level of introgression signal is tied to the geographic proximity of the species involved and the amount of past environmental change in the area in which they occur. This study confirms the utility of large, comprehensively sampled phylogenomic datasets for examining the geography of introgression and its causes. Further, our results provide an early indication that the degree of hybridization and introgression is dynamic and that this variation might reflect deterministic impacts of geography and environment on how diversity evolves.

Although historically characterized as the “grossest blunder […] we can conceive of an animal making” (Fisher 1930), we now know that hybridization is rampant across the animal tree of life (Mallet et al. 2016). In some cases, hybridization moves genetic variation from one species into another, a process known as introgression or introgressive hybridization (Harrison and Larson 2014). Introgression spans a continuum, from the selective introgression of a single allele (Lamichhaney et al. 2016) to the sustained exchange of genes across a hybrid zone (Barton and Hewitt 1985) or the near‐total merger that occurs following reticulation into a hybrid species (Mallet 2007). Hybridization can have fundamental and opposing effects on evolution. On the one hand, hybridization can serve as a source of novelty (Anderson 1953; Stebbins 1959; Lewontin and Birch 1966) through adaptive introgression of alleles across species boundaries (Pardo‐Diaz et al. 2012; Racimo et al. 2015), by reinforcing existing reproductive barriers (Lukhtanov et al. 2005), or by producing new hybrid species (Mallet 2007). This creative role for hybridization may explain observed correlations between hybridization frequency and net species diversification, as seen in salamanders and plants (Mitchell and Whitney 2021; Patton et al. 2020). In contrast, and perhaps more commonly, hybridization can be a destructive force. Hybrid individuals often have lower fitness than their parents, both because their genomes contain genetic incompatibilities and because admixed phenotypes can be mismatched to the local environment (Barton and Hewitt 1985). At its extreme, hybridization can lead to species extinction through genetic or demographic swamping, ultimately eroding species diversity (Todesco et al. 2016; Vonlanthen et al. 2012).

Differences in the frequency of hybridization among organismal groups or geographic regions may provide a window into evolutionary differences underlying broad biodiversity patterns. Not all species are equally likely to hybridize. Species vary both in the rates at which key traits affecting reproductive isolation evolve, as seen for bird song (Weir and Wheatcroft 2011) and pollination syndrome (Wessinger et al. 2019), and in the rate at which reproductive isolation evolves (Rabosky and Matute 2013). Hybridization will likely occur less often in species groups that evolve reproductive barriers more quickly. The biogeographic and environmental contexts in which speciation occurs matters, as well. Species that originate close to each other—for example, lake flocks of African cichlids (Meier et al. 2017)—have more opportunities for hybridization (Hamlin et al. 2020). Further, species that originate in variable environments, like the historically unstable regions of the temperate zone (Hewitt 2004), tend to have more dynamic ranges that might lead to secondary contact between species before reproductive barriers have evolved (Cutter and Gray 2016). Together, these verbal arguments suggest that when and where hybridization occurs might be predictable based on both their intrinsic properties and the geographic and environmental context in which they form (Dagilis et al. 2021; Hamlin et al. 2020; Leighton et al. 2021; Mitchell et al. 2019).

Characterizing hybridization dynamics requires methods for comparably measuring hybridization across the tree of life. Identifying hybrids based on phenotypic data is typically challenging unless hybridizing populations are conspicuously different and hybrids formed recently (Mallet 2005). Given genetic data from just a few loci, recent hybrids can be more robustly identified across a wider range of species (Anderson and Thompson 2002). However, as the hybridization event recedes in the past, its signature decays through the effects of recombination, selection, and drift (Sedghifar et al. 2016). Fortunately, the growth of genome‐scale datasets, combined with new analytical approaches (Payseur and Rieseberg 2016), have made it easier to identify the signal of introgressive hybridization across diverging genomes. When combined with phylogenetic hypotheses and biogeographic information (Folk et al. 2018; Pease et al. 2016; Suvorov et al. 2021), these approaches can allow comparative inference of introgression across multiple species.

One way that introgression manifests in genomic datasets is via gene tree discordance, or when evolutionary relationships inferred from independent genetic loci conflict with the species tree (Degnan and Rosenberg 2009). Gene tree discordance is straightforward to measure but determining its origins can be challenging. Discordance has multiple sources, including incomplete lineage sorting, paralogy, and gene tree estimation error as well as introgression (Degnan and Rosenberg 2006; Maddison 1997; Roch and Warnow 2015). The degree to which introgression contributes to the rampant gene tree discordance seen across phylogenomic datasets relative to these other sources is poorly understood (Degnan and Rosenberg 2009 but see Knowles et al. 2018; Pease et al. 2018; Bravo et al. 2019). Strategies are available, however, to identify cases of gene tree discordance putatively stemming from introgression. For example, given a species triad and outgroup, Patterson's *D*‐statistic (also known as the “ABBA‐BABA” test) compares the relative frequency of ABBA sites (in which species 2 and 3 share derived alleles) versus BABA sites (in which species 1 and 3 share derived alleles; Green et al. 2010; Durand et al. 2011). Under incomplete sorting, the numbers of ABBA and BABA sites are expected to be similar, but introgression leads to an imbalance in the two types of sites, the extent of which is captured by the *D*‐statistic. The application of such approaches to comprehensive phylogenomic datasets has the potential to provide a new perspective on introgression dynamics across the tree of life (Edelman et al. 2019; Suvorov et al. 2021).

In this study, we use a phylogenomic dataset of 2389 loci sampled from nearly all 1306 species in the suboscine bird radiation to examine signatures of hybridization and introgression across lineages and geographic areas. Suboscines are perching birds of the suborder Tyranni, which originated within the last ∼50 million years (Harvey et al. 2020; Oliveros et al. 2019; Prum et al. 2015). Suboscine diversity is centered in the Neotropics, but the group occurs throughout North and South America and in the Afrotropics and Indomalayan/Australasian regions. Suboscines are a morphologically, ecologically, and behaviorally diverse clade that includes the primarily insectivorous and variously migratory tyrant flycatchers (Tyrannidae), frugivorous manakins (Pipridae) with lek mating systems, mouse‐like tapaculos (Rhinocryptidae) of the forest understory, brilliantly colored cotingas (Cotingidae) and broadbills (Eurylaimidae, Calyptomenidae), and cryptically patterned ovenbirds (Furnariidae) and woodcreepers (Dendrocolaptidae; Stotz et al. 1996). Information on hybridization in the group remains fragmentary (Graves 1992), although hybrids involving over 100 suboscine species have been recorded (McCarthy 2006).

We first use the full suboscine tree to characterize variation in gene tree discordance among lineages and geographic regions. We then estimate variation in the signal of introgression across the set of all possible, exclusive species triads of suboscines (*n* = 206, Fig. 1) using a topological approach (Patterson's *D*‐statistic). Finally, we ask two related questions: to what extent is gene tree discordance determined by introgression signal, and is introgression signal predictable based on geography, environment, and genetic divergence between hybridizing taxa? To address these questions, we test for associations among estimates and key predictor variables within a causal modeling framework.

**Figure 1:**
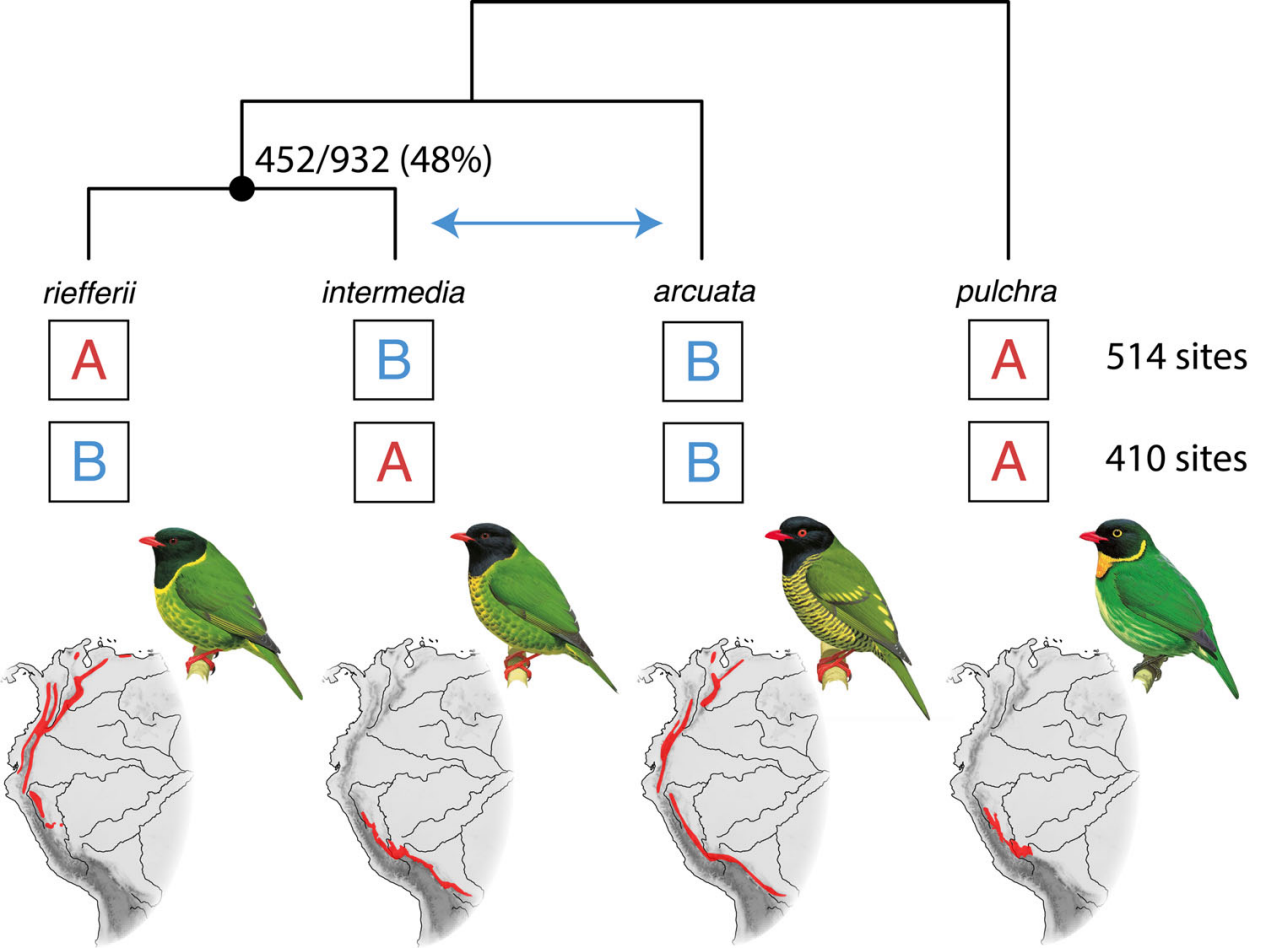
Exemplar triad and outgroup showing the four‐tip phylogeny, level of gene tree discordance, number of ABBA and BABA sites, and images of the birds and their geographic ranges. This triad is in the genus *Pipreola* (Cotingidae) and has a significant *D*‐statistic (250‐site subsample: *D* = 0.244, *Z* = 3.895, *P* = 9.81 × 10^–5^) indicating elevated sharing of alleles between the species *intermedia* and *arcuata* (ABBA site pattern). Gene tree discordance (number of discordant gene trees over number of resolved discordant and concordant gene trees) is shown for the node subtending the sister species; introgression among triad species will most likely increase discordance at this node. The counts of ABBA and BABA sites are based on the allele frequency approach, which accounts for nonfixed alleles within a species. Representative images are included for each species (illustration © Lynx Edicions).

## Methods

### STUDY SYSTEM AND SAMPLING

Our sampling leverages the phylogenomic dataset of suboscine bird species published by Harvey et al. (2020). This comprehensive phylogenomic dataset contains 98.2% (1283) of nominal suboscine bird species sampled at 2389 genomic loci. The targeted genomic regions comprise two sets of highly conserved loci: 2321 ultraconserved elements (Faircloth et al. 2012) and 96 exons commonly used in avian phylogenomics (Harvey et al. 2017). Briefly, these loci were sampled using a target‐capture approach, followed by de novo assembly of raw reads and mapping of assemblies to targeted loci for annotation (full details available in Harvey et al. 2020).

### MEASURING LEVELS OF GENE TREE DISCORDANCE

To characterize levels of gene tree discordance across the suboscine phylogeny, we compared inferred gene trees to the consensus tree. The consensus tree was taken from Harvey et al. 2020 and is a rooted ExaML concatenated tree based on the larger, minimally filtered alignment matrix and time calibrated using a penalized likelihood approach (Fig. S1). The tree was trimmed to species based on existing taxonomies (Chesser et al. 2017; Remsen et al. 2011). Per locus, we used IQ‐TREE version 1.6.12 to infer gene trees under a GTRGAMMA model (Minh et al. 2020) and to calculate Shimodaira‐Hasegawa‐like (SH‐aLRT) support scores for each node (following Guindon et al. 2010). When nodes have low support, this can reflect low information content, which can lead to gene tree estimation error and increases in gene tree discordance (Blom et al. 2017; Roch and Warnow 2015). Given that we are not interested in the role of gene tree estimation error in gene tree discordance, we collapsed nodes with <80 SH‐aLRT support before calculating gene tree discordance. We compared these gene trees to the consensus tree using phyparts (Smith et al. 2015). Phyparts calculates the number of gene trees that are concordant versus discordant across each node on the consensus tree. For a gene tree to be concordant at a given node, the node must have the same descendants as the comparable node in the consensus tree, excepting missing taxa. We calculated levels of gene tree discordance as the ratio of discordant gene trees to the total number of resolved (concordant + discordant) gene trees at each node. Because geographic distribution data are not available for the nodes at which discordance is measured, we mapped the geography of gene tree discordance by obtaining a time‐integrated estimate of the gene tree discordance in the lineage leading to each extant species with range data. Specifically, we calculated a weighted average of the gene tree discordance at all nodes subtending a given species, down‐weighted according to its node depth in the tree, analogous to the strategy used to calculate the DR statistic for diversification (Jetz et al. 2012; Title and Rabosky 2019).

### MEASURING LEVELS OF INTROGRESSION

There are two primary approaches to measuring introgression across multiple species pairs. The first is to infer phylogenetic networks, in which introgression across species is explicitly modeled in determining evolutionary relationships (e.g., PhyloNetworks, Solís‐Lemus et al. 2017). The second compares topologies across variants (Durand et al. 2011) or across gene trees (Suvorov et al. 2021); imbalances across topologies indicate introgression. Here, we apply the topological approach because it is more tractable for the broad phylogenetic scale of this study. In particular, we calculate the *D*‐statistic (Durand et al. 2011), which measures in a four‐tip pectinate phylogeny the relative ratios of “ABBA” versus “BABA” topologies across variable sites. If the two topologies are relatively equal, no introgression is inferred; imbalances toward either topology suggest either introgression between species 1 and species 3 (“BABA”) or between species 2 and species 3 (“ABBA”).

To calculate the *D*‐statistic, we first identified species triads in the species phylogeny. Previous studies have fruitfully taken an inclusive approach and measured the *D*‐statistic across all possible triads in the consensus tree, including paraphyletic triads (Malinsky et al. 2018). Disentangling historical introgression from more recent introgression can be challenging in this approach (Pease et al. 2016). Thus, we instead examined only those triads representing monophyletic groups of extant species, in total identifying 206 possible triads (Fig. S1). We then identified the most‐closely related outgroup; where there were multiple possible outgroups, we selected the species for which we had the most complete locus‐level assembly.

For each species group, we performed a set of population genomic analyses to measure the *D*‐statistic (Fig. 1). To do so, we needed to call variants across all species of interest with respect to a common set of reference loci. Thus, we used an iterative reference‐based approach in which raw reads from all targeted species were mapped to the same reference (Sarver et al. 2017), in this case the assembled locus set for the outgroup species. Per species, we first trimmed raw reads of adaptor sequence using Trimmomatic version 0.39 (Bolger et al. 2014) and collapsed overlapping reads using PEAR version 0.9.11 (Zhang et al. 2014). We mapped trimmed reads to the reference using bwa version 0.7.17 (Li 2013), called variants using bcftools version 1.10.2 (Li 2011), and then filtered to retain only high‐coverage and high‐quality variants (coverage >20×, quality >20). We then mutated the original reference to include these variants and repeated this process three more times. The reference acquires all the nucleotide substitutions specific to the species through these multiple rounds of variant calling. Using each species’ mutated reference , we then called variant and invariant sites using bcftools and only retained those sites with coverage >5× and quality >20. Our initial data exploration found that the number of sites used to calculate the *D*‐statistic affected the likelihood that the test was significant (Fig. S2). Accordingly, we randomly subsampled all datasets across all species triads to 100, 250, or 500 ABBA‐BABA informative sites. Not all triads could be included across all datasets because they were sampled at too few informative sites. Using these filtered and subsampled sites, we calculated the allele frequency‐based *D*‐statistic across each species triad (Durand et al. 2011). We then converted our *D*‐statistic to a *Z*‐score by dividing it by the standard deviation of the *D*‐statistic across 100 bootstraps (Eaton and Ree 2013). We used this *Z*‐score to assess significance, with *P*‐values <0.05 as significant.

### MODELING PREDICTORS OF INTROGRESSION AND GENE TREE DISCORDANCE

We performed causal modeling to explore how incomplete lineage sorting (ILS) and introgression contribute to gene tree discordance and to evaluate associations with key organismal and evolutionary factors that might predict ILS and introgression (Fig. S3). ILS is expected to be highest under a few demographic scenarios: when splitting times are short, ancestral population sizes are large, and ancestral populations are subdivided (Maddison and Knowles 2006; Slatkin and Pollack 2008). To account for these demographic factors, we included range size as a proxy for population size (Gaston 2003) and internode lengths between taxa. We calculated range sizes using range maps from the Birds of the World (BirdLife International and NatureServe 2018).

There are a few scenarios in which we might expect introgression to be more likely. First, introgression is more likely between species with shallower divergences that have yet to evolve reproductive barriers (Coyne and Orr 1989; Pulido‐Santacruz et al. 2020; Sasa et al. 1998; Singhal and Bi 2017); accordingly, we included divergence time between the hybridizing taxa based on the consensus tree from Harvey et al. (2020). Second, if two species overlap geographically, then they have more opportunity to mate with each other, leading to introgression. To include this factor, we measured geographic distance as the closest distance between the edges of current geographic ranges of the hybridizing taxa. Under this metric, both narrowly parapatric and sympatric species have zero distance. Third, if two species live in historically unstable environments, then their geographic ranges might be dynamic through time, leading to secondary contact and thus introgression (Cutter and Gray 2016). As proxies for range stability, we used latitude and the climate change velocity in average temperature across the ranges of the hybridizing taxa (Loarie et al. 2009). Lower latitudes are thought to be more stable (Fischer 1960); to include this in our model, we averaged the centroid latitude of the geographic ranges of the two hybridizing species. For climate change velocity, we calculated the change between present and at the Last Glacial Maximum (21,000 before present) using the CHELSA version 1.2 models (Karger et al. 2017). Other factors, in particular intrinsic biological traits of species, may also be important predictors of the probability of introgression, but were not a focus of this study.

To determine how these factors impact introgression, ILS, and thus gene tree discordance, we used a phylogenetic path analysis implemented via the R package phylopath version 1.1.2 (van der Bijl 2018). We constructed four models: a null model in which gene tree discordance only results from ILS‐related factors, a model in which gene tree discordance only results from introgression‐related factors, and a model that included both ILS and introgression‐related factors (Fig. S4). Per triad, we used the gene tree discordance at the node subtending the sister species (see Fig. 1); discordance at this node reflects variance in phylogenetic relationships among the three triad species. To include introgression in our model, we used the *D*‐statistic as an estimate of introgression strength. However, because the *D*‐statistic does not always accurately infer introgression strength (Hibbins and Hahn 2021; Martin et al. 2015), we repeated our analysis with introgression coded as a binary variable (present or absent) based on the *Z*‐score for the *D*‐statistic and an ɑ = 0.05. We compared these models using the *C*‐statistic information criterion corrected for small samples (CICc). Prior to modeling, we took the absolute values for latitude and climate change velocity, and we took the natural log of all variables but gene tree discordance. We ran this analysis across the three subsampled datasets of 100, 250, or 500 ABBA‐BABA informative sites.

Finally, to account for phylogenetic uncertainty, we re‐ran our analyses across a bootstrap distribution of concatenated trees and across a coalescent‐based topology. See *Methods* in the Supporting Information for details.

## Results

**Figure 2:**
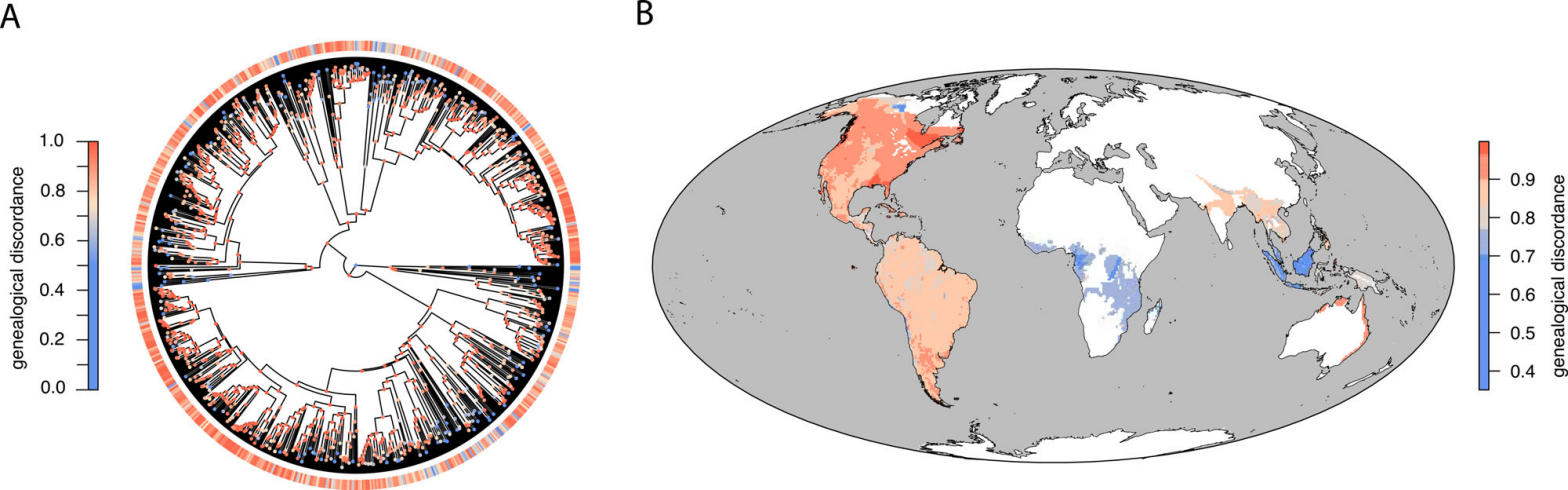
Variation in levels of gene tree discordance. **(A)** Gene tree discordance varies among nodes of the suboscine phylogeny and across species, based on a time‐integrated summary of the history of gene tree discordance in their subtending lineages. Nodes are colored according to the proportion of discordant gene trees, whereas summary discordance values for species are depicted as colored bands in the encircling ring. **(B)** The average level of gene tree discordance subtending the species occurring in a region varies geographically. The map is based on an equal‐area projection with 200‐km^2^ grid cells. Color scales differ between the two panels for ease of interpretation.

Across nodes on the suboscine tree, between 11.7% and 100% (mean = 85.4%) of resolved gene trees were discordant with the consensus tree (Fig. 2A; Dataset S1). Eleven nodes had the maximum value of gene tree discordance (100%), five of which were in one tyrant flycatcher clade (Contopini + Xolmiini) that contains most of the suboscines occurring at high latitudes. Two other nodes with the maximum discordance value were in the tyrant flycatcher genus *Myiarchus*, whereas the others were deeper nodes early in the Tyrannidae (2), Thamnophilidae, and Furnariidae (2) families. The lowest values of gene tree discordance were at nodes within the genera *Hylopezus* (Grallariidae), *Melanopareia* (Melanopareiidae), *Pitta* (Pittidae), *Neodrepanis* (Philepittidae), and *Cinclodes* (Furnariidae) and were in parts of the phylogeny typified by longer branches. When mapped across space, the average gene tree discordance subtending the species occurring in each 200‐km^2^ grid cell ranged between 35.1% and 99.8% (mean = 85.6%; Fig. 2B).

**Figure 3:**
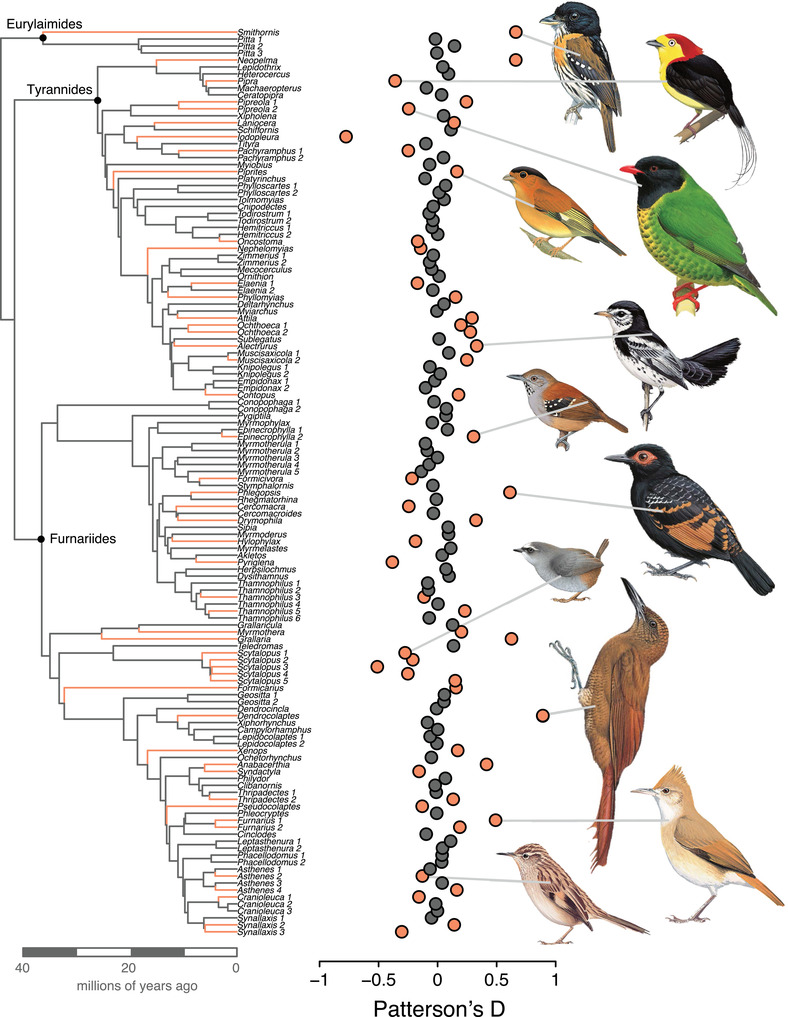
Phylogeny of the 130 species triads used in the primary dataset; each triad is represented by one constituent tip and labeled by the genus name for the triad. The three suboscine infraorders are labeled at their crown nodes. Mapped across the phylogeny are the *D*‐statistic values for each triad. Triad branches and points that have *D*‐statistics significantly different from zero (*P* < 0.05) are shown in orange. In total, 49 of 130 (38%) species triads have significant *D*‐statistics, suggesting introgression has been fairly common across the suboscine radiation. Species from select triads with significant *D*‐statistics are represented by images (illustration © Lynx Edicions).

We were able to infer the *D*‐statistic for 203 out of 206 triads for an average of 450.5 ABBA‐BABA informative sites across an average of 325 loci (Fig. S5); the other three triads were dropped due to low data recovery. Because the number of informative sites used affects the likelihood of generating a significant *D*‐statistic (Fig. S2), we created three subsampled datasets: 100 sites (*n* = 179 triads), 250 sites (*n* = 130 triads; Table S1), and 500 sites (*n* = 63 triads). All subsampling schemes returned highly correlated estimates of the *D*‐statistic (*r* = 0.89–0.97; Fig. S6), so for brevity, we focus on the 250 sites subsampling scheme. Across this dataset, the *D*‐statistic ranged from –0.78 to 0.89 (Fig. 3), and 49 of 130 triads (38%) had a significant *D*‐statistic (*P* < 0.05). The highest five *D*‐statistics were in *Dendrocolaptes* woodcreepers (Dendrocolaptidae), *Smithornis* broadbills (Eurylaimidae), *Neopelma* manakins (Pipridae), *Grallaria* antpittas (Grallariidae), and *Phlegopsis* antbirds (Thamnophilidae) and the lowest were in *Iodopleura* purpletufts (Tityridae), *Scytalopus* tapaculos (Rhinocryptidae), *Pyriglena* antbirds (Thamnophilidae), *Pipra* manakins (Pipridae), and *Synallaxis* spinetails (Furnariidae; see Table S1).

**Figure 4:**
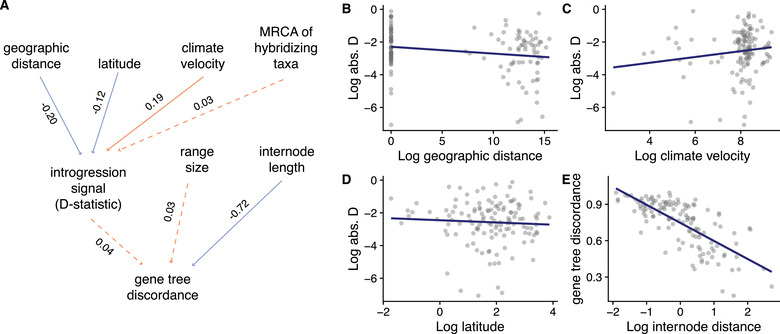
(A) Best fitting model from phylogenetic path analysis modeling predictors of introgression and gene tree discordance. In this model, various factors predict introgression signal. In turn, introgression signal and additional factors predict gene tree discordance. Shown are standardized correlation coefficients. Orange and blue arrows indicate positive versus negative correlations, respectively; solid and dotted arrows indicate significant versus nonsignificant correlations, respectively. As predicted, (B) shorter geographic distances between species and (C) greater climate change velocity lead to greater introgression signal (as measured by *Z*‐scores for the *D*‐statistic), and (E) shorter internode distances (e.g., faster splitting times) lead to greater levels of gene tree discordance. Contrary to predictions, higher latitudes lead to reduced introgression signal.

Our best model for predictors of gene discordance included factors that are thought to affect both ILS and introgression, as opposed to models that included only ILS‐related or only introgression‐related factors (Fig. S4). In particular, four factors emerged as significant. As predicted, introgression was more common in geographically proximate species and in species with less stable ranges. Geographic distance was negatively correlated with introgression signal (*r* = –0.20; Fig. 4B), and climate change velocity was positively correlated with introgression signal (*r* = 0.19; Fig. 4C). Given that higher latitudes are less stable, we expect to see more introgression at higher than at lower latitudes. Contrary to this expectation, latitude was negatively correlated with introgression signal (*r* = –0.12; Fig. 4D). Finally, as predicted under ILS, internode length was negatively correlated with levels of gene discordance (*r* = –0.72; Fig. 4E). However, introgression was not a significant contributor to gene tree discordance (Fig. 4A). These findings were generally similar when treating introgression signal as a binary variable (Fig. S7) and when using alternate subsampling schemes and accounting for phylogenetic uncertainty (see *Results* in the Supporting Information; Tables S2 and S3).

## Discussion

### RAMPANT SIGNALS OF DISCORDANCE AND INTROGRESSION

Our analyses suggest that both gene tree discordance and introgression are rampant across the suboscine radiation. Gene tree discordance is so common that some nodes in the consensus phylogeny estimated from concatenated sequences are not present in a single underlying gene tree (Fig. 2A). The high estimates of gene tree discordance in this dataset likely have a few underlying causes. First, our measure of gene tree discordance is both binary and stringent. Gene tree bipartitions must perfectly correspond to those in the consensus tree such that a single misplaced tip qualifies as discordance. Alternate metrics for gene tree discordance, such as those based on calculating likelihood support across competing topologies (Shen et al. 2017; Smith et al. 2020), may offer more nuanced and sensitive estimates of gene tree conflict but are less tractable with larger datasets. Second, although we attempted to account for gene tree estimation error by removing topological relationships with low nodal support, the low information content of the loci examined may provide opportunities for isolated homoplasy or sequencing errors to overwhelm phylogenetic signal. Third, high levels of gene tree discordance are possible if the consensus tree contains erroneous relationships. Fourth, the rapid radiation of suboscines since the group's origin (Harvey et al. 2020) has likely provided opportunities for incomplete lineage sorting. Our models show that levels of gene tree discordance increase as internode distances between species decrease (Fig. 4E). This pattern accords well with predictions that gene tree conflict should be the expectation when internal branches are short (Degnan and Rosenberg 2006), and our results are consistent with data from other rapid radiations demonstrating high levels of gene tree discordance (Cloutier et al. 2019; Pease et al. 2016; Roycroft et al. 2020; Suh et al. 2015).

Widespread introgressive hybridization is another factor that could drive the overall high levels of gene tree discordance. We note that we did not find a significant relationship between variation in introgression signal and gene tree discordance (Fig. 4A). However, there was a weak positive relationship that suggests a relationship may be identified with a larger sample. The *D*‐statistic results indicate that 38% of triads show signals of introgression. Historically, suboscine birds were thought to exhibit relatively low hybridization rates compared to other birds (Cadena et al. 2007; Graves 1992). More recently, hybridization and introgression have been found in several suboscine species (Brumfield et al. 2001; Pulido‐Santacruz et al. 2020; Weir et al. 2015). Birds, in general, hybridize at relatively high rates; about 16% of avian species are known to hybridize in nature based on data from a recent meta‐analysis (Ottenburghs et al. 2015). An increasing number of focused studies indicate that introgression is also prevalent in many avian systems (Beckman et al. 2018). Frequent hybridization and introgression in birds may be associated with their high dispersal abilities relative to other animals, leading to more frequent encounters between heterospecifics or more rapid acquisition of secondary contact. Further, although birds are well‐known for their spectacular evolution of plumage and song diversity, divergence in these premating signals does not always prevent interspecific and even intergeneric matings (Hudson and Price 2014; Marini and Hackett 2002; Parkes 1961; Price 2008; Uy et al. 2018). Finally, compared to other vertebrates, birds evolve postzygotic isolation relatively slowly (Prager and Wilson 1975; Price and Bouvier 2002; Fitzpatrick 2004), permitting postdivergence gene flow.

Our estimates of introgression signal for suboscines are not outstanding, however, relative to those estimated using phylogenomic data from other animal and plant groups. The 38% frequency of introgression in suboscine triads is comparable to rates seen in groups within mammals, fish, plants, and corals (Escudero et al. 2014; Ferreira et al. 2021; Hamlin et al. 2020; Jónsson et al. 2014; Malinsky et al. 2018; Quattrini et al. 2019; Vargas et al. 2017). Many of these studies identified multiple cases of introgression within modestly sized clades; for example, Jónsson et al. found evidence for four introgression events in a group of nine equid species and subspecies. Many cases of introgression are between populations or subspecies (e.g., Irwin et al. 2018; Malinsky et al. 2018); however, recent studies in primates and *Drosophila* uncovered multiple instances of older introgression, including between species that diverged ∼20 million years ago (Suvorov et al. 2021; Vanderpool et al. 2020). For comparison, in our study, hybridizing species diverged anywhere from 1.1 to 18.2 million years ago, spanning the full range of divergence times seen among sampled triads. The average divergence time among hybridizing species was 4.3 million years (Fig. S8). By examining a broad phylogenetic group, we show that previous observations of frequent introgression across both recently diverged and older species pairs hold across a large set of species. Our results strongly support the emerging consensus that introgression is pervasive across the tree of life (Mallet et al. 2016).

### CAUSES OF VARIATION IN INTROGRESSION SIGNAL

We found that both gene tree discordance and introgression signal varied across lineages and space (Figs. 2, 3, and S9). Nodes with the highest gene tree discordance were concentrated in rapidly diversifying clades, including some (Contopini + Xolmiini, *Myiarchus*) that occur at higher latitudes (Fig. 1; Dataset S1). Geographic variation in gene tree discordance resembles the map of speciation rates for suboscines (Harvey et al. 2020), in which we see less discordance and lower speciation rates in tropical hotspots of species diversity. This correlation may arise because faster speciation leads to shorter internodes and shorter internodes result in elevated gene tree discordance (Fig. 4E). However, this pattern likely captures the impact of elevated incomplete lineage sorting in these parts of the tree as much as it does the role of introgression.

The variation observed in Patterson's *D*‐statistic may better reflect dynamics in the processes of hybridization and introgression. The triads with the highest introgression signal were scattered across the phylogeny (Fig. 3; Table S1). Future work incorporating data on the intrinsic traits of these lineages may reveal impacts of their diverse biologies on gene tree discordance and introgression, but the focus of this work was on geographic and environmental predictors.

Variation in introgression signal was associated with three variables related to the geography and the environment of the lineages examined. Elevated signals of introgression were observed in lineages that were closer geographically, that experienced elevated rates of climate change through time, and that occurred at lower latitudes. The link between introgression signal and geographic proximity is intuitive: species that are closer in space will have more opportunities for interspecific matings, which is perhaps best illustrated by introgression across zones of secondary contact (Harrison and Larson 2014) and hybridization following anthropogenic displacement of species (Todesco et al. 2016). However, current range boundaries do not always reflect historical boundaries, and introgression events are often inferred between currently geographically distinct taxa (Folk et al. 2018; Manthey et al. 2020; Pulido‐Santacruz et al. 2020; Vanderpool et al. 2020). Twenty‐six of our 49 hybridizing pairs have overlapping ranges, and hybridizing pairs with greater overlap do not show more evidence for introgression (Fig. S10). The remaining 23 hybridizing pairs occur an average of ∼700 km apart. Hybridization between nonoverlapping species might reflect shifting geographic ranges through time and the high dispersal capacity of birds. Contrary to other studies (Dagilis et al. 2021; Hamlin et al. 2020), we find no evidence for increased introgression between more recently diverged taxa (Fig. 4A).

The positive association between introgression signal and rates of climate change through time fits well with a verbal model in which dynamic environments may regularly reshuffle newly formed species across regions. In some instances, this can lead to species loss through fusion (“ephemeral ecological speciation”; Cutter and Gray 2016; Rosenblum et al. 2012; Schluter 2016). Although introgression from extinct species would be difficult to detect based on data from extant species (but see Durvasula and Sankararaman 2020), elevated rates of hybridization even in species that persist are still consistent with this model (Cutter and Gray 2016). Birds at high latitudes (and presumably in more dynamic environments) also evolve premating reproductive isolation and sympatry faster than those at low latitudes (Mason et al. 2017; Weir and Price 2011; Weir and Wheatcroft 2011). In contrast, postzygotic reproductive isolation may be more important in the tropics. Tropical *Drosophila* evolve postzygotic isolation at higher rates than their temperate counterparts (Yukilevich 2013), and recent results suggest that postzygotic barriers may play a significant role in limiting gene flow between tropical bird species (Pulido‐Santacruz et al. 2018, 2020). Introgressive hybridization may be more limited during tropical speciation due to the presence of postzygotic isolation but more frequent in high latitudes if species rapidly come into sympatry and the premating reproductive isolation mechanisms they have evolved are imperfect.

Surprisingly, the signal of introgression is positively associated with environmental change but negatively associated with latitude. Higher latitudes generally exhibit more dynamic environments, as seen in our data (*r* = 0.40; Fig. S11). However, the latitude‐introgression association was particularly inconsistent across subsampled datasets (Table S2), suggesting it is not an exceptionally robust result. The small number of temperate suboscine species may have limited our power to detect a relationship between introgression and latitude. Further, the covariation between latitude and climate instability might have made it difficult to distinguish a latitudinal trend. Finally, perhaps tropical environments provide more opportunities for introgression, both because the high species richness means many closely related species co‐occur and because many tropical environments may be more dynamic than is often appreciated (Baker et al. 2020).

### SOURCES OF UNCERTAINTY

Our approach makes some simplifying assumptions that might explain the overall low explanatory power of our model in accounting for sources of variation in introgression extent (Fig. 4; Table S2). First, we do not account for all possible occurrences of introgression. We modeled introgression as occurring between species in exclusive triads. The average crown age of these triads was 4.3 Ma (Fig. S8). Given that viable hybrids can form in birds over >55 Ma (Price and Bouvier 2002) and that many closely related suboscine bird species occur in the same geographic areas, there likely was unmodeled introgression across broader phylogenetic scales. Under this scenario, where introgression occurs from unsampled “ghost” populations (Pease and Hahn 2015), we might underestimate the overall prevalence of introgression. Unmodeled introgression between ancestral species might also bias our understanding of the frequency and nature of introgression (Malinsky et al. 2018; Suvorov et al. 2021), for example, by leading us to infer multiple cases of current introgression among daughter species (Pease et al. 2016). Such historical introgression might be widespread in rapidly radiating species groups such as the suboscines, where young lineages are more likely to interact before evolving reproductive isolation (Edelman et al. 2019; Meyer et al. 2017). On a related note, our analyses used the currently accepted taxonomy to infer introgression. Including unrecognized diversity, such as subspecies that might merit species status (e.g., Dickens et al. 2021), can yield additional instances of introgression.

Second, methods for estimating introgression can be prone to both false positives and negatives. The *D*‐statistic is a robust approach to inferring introgression (Zheng and Janke 2018; Kong and Kubatko 2021 but see Hibbins and Hahn 2021), even in cases of limited individual and genomic sampling. Still, the *D*‐statistic has limitations. By design, the *D*‐statistic cannot identify cases of introgression between sister species. Also, high levels of incomplete lineage sorting—that is, in cases where the distance between splitting times is short and population sizes are large—can decrease the power of the *D*‐statistic to identify cases of introgression (Zheng and Janke 2018). In suboscine birds, where splitting times are rapid, incomplete lineage sorting is likely pervasive, as our estimates of gene tree discordance suggest (Fig. 2). In addition, because we used a subset of the genome and the dataset includes only one individual per species, we likely missed cases where a small fraction of the genome was exchanged, where introgression was spatially structured, or where introgression events are old (Good et al. 2015; Yu et al. 2011). As such, the cases that we did identify likely represent diversification histories where introgression was prolonged and widespread geographically. The *D*‐statistic also can be prone to misidentifying cases of introgression when ancestral populations have significant structure (Slatkin and Pollack 2008). Because tropical bird species tend to show more population structure than temperate bird species (Smith et al. 2017), this bias is unlikely to lead to the reduced introgression in stable regions we observed (Fig. 4C), but could explain why we see weak evidence of more introgression at lower latitudes compared to higher latitudes (Fig. 4D). Despite its limitations, the *D*‐statistic remained the best available approach for inferring introgression in our study, given the scope of our genomic and species‐level sampling. Future studies with expanded individual and genomic sampling could fruitfully explore alternative approaches to inferring introgression (as reviewed in Hibbins and Hahn 2021).

Third, our predictors for variance in introgression signal could be expanded considerably. Given the broad comparative scope of our study, our predictors are necessarily simplistic. For example, as a proxy for the strength and extent of reproductive barriers, we used the divergence time between hybridizing species. Although divergence time strongly predicts the strength of reproductive barriers (Coyne and Orr 1989), levels of mating isolation and hybrid inviability would serve as more nuanced and possibly better proxies. Additionally, we did not consider any species‐level traits that might impact the likelihood of introgression. Traits such as dispersal distance, which could affect the frequency of interspecific interactions, or plumage or song divergence, which could be proxies for mating behavior, might more strongly predict introgression (Winger 2017). Further, our predictors focused on hybridization factors, but our metrics of introgression do not necessarily capture all hybridization. If selection is strong against hybrids, whether due to intrinsic or extrinsic factors, then introgression rates can be low even if hybridization occurs frequently (Epling 1947). Additionally, the factors that control introgression dynamics might be too dynamic and variable to result in concerted patterns across the broad phylogenetic scale of this study. These limitations of our approach might explain why our model explains a relatively modest amount of the variation in introgression signal (Fig. 4).

### FUTURE DIRECTIONS

Our data suggest that hybridization and introgression are prevalent in suboscine birds but vary across the radiation. To understand the impact of these processes on suboscine evolution, we need to identify more accurately when introgression occurred between hybridizing taxa and what portions of the genome were exchanged. Because our genomic sampling was limited, we were unable to estimate the extent of introgression across the genome, which likely affects the evolutionary trajectory of the species. However, even restricted introgression can have a significant impact. Single introgressing loci can drastically affect individual fitness both negatively—for example, as seen in Dobzhansky‐Muller incompatibilities (Powell et al. 2020)—and positively—for example, as seen in cases of adaptive introgression (Lamichhaney et al. 2018). Characterizing the nature of introgressed genetic material—including its spatial context and phenotypic impacts—will allow us to determine how hybridization affects speciation and will help us determine when and how hybridization acts as a driver versus a brake on evolution.

## AUTHOR CONTRIBUTIONS

SS and MGH conceived and designed the study. All authors compiled data. SS and MGH analyzed data. All authors interpreted the results and wrote the manuscript.

## DATA ARCHIVING

No new data were generated for this study. Code used in analysis and figure generation and databases containing results are available at: https://github.com/singhal/bird_hyb.

## Supporting information


**Dataset S1**: Levels of gene tree discordance across the concatenated, maximum likelihood suboscine phylogeny (1286 tips).
**Table S1**: Species triads used in the focal analyses: 250‐sites species test (*n* = 130 triads).
**Table S2**: Standardized regression coefficients as estimated by phylogenetic path analysis across subsampling schemes: 100‐sites subsampling (*n* = 179 triads), 250‐sites set (*n* = 130), and 500‐sites set (*n* = 63).
**Table S3**: Standardized regression coefficients as estimated by phylogenetic path analysis across 100 bootstrap trees for the 250‐sites data set (*n* = 130 triads).
**Figure S1**: Phylogenetic distribution of the triads originally selected for this study (shown in blue) and triads eventually retained in the primary data set (shown in orange) on the 1286‐species phylogeny of suboscine birds.
**Figure S2**: The number of ABBA‐BABA informative sites sampled affects the likelihood of inferring a significant *D*‐statistic.
**Figure S3**: Model for how different organismal and evolutionary factors influence levels of gene tree discordance.
**Figure S4**: Three of the four models fit to our dataset using phylogenetic path analysis.
**Figure S5**: The (A) number of *D*‐statistic informative SNPs and (B) and number of loci in which they fall across the 203 triads for which we could infer the *D*‐statistic.
**Figure S6**: Correlations in *D*‐statistic estimates across different subsampling strategies.
**Figure S7**: Results from model fitting in which introgression signal is treated as a binary variable (0 = no evidence for introgression, 1 = evidence for introgression).
**Figure S8**: Descriptions of the triads originally selected for this study (*n* = 206; shown in blue) and triads eventually retained in the primary data set (*n* = 130; shown in orange): (A) Internode length of triad in millions of years (myr), (B) age of most recent common ancestor (MRCA) of triad, and (C) age of most recent common ancestor (MRCA) of triad plus the outgroup.
**Figure S9**: Maps of the distributions of triads (A) with and (B) without significant *D*‐statistics.
**Figure S10**: Correlation between log geographic range overlap and log absolute *D*‐statistic of sympatric hybridizing pairs (*n* = 26).
**Figure S11**: Correlation between absolute latitude and log absolute climate instability of triads in the 250 ABBA‐BABA site dataset (*n* = 129).
**Figure S12**: Variation in levels of gene tree discordance across a coalescent‐based ASTRAL species tree.

Supplementary information
