# Supplementary material for: The dynamics of introgression across an avian radiation
Source: Evol Lett. 2021 Sep 28;5(6):568–81. doi: 10.1002/evl3.256 (PMC8645201; doi:10.1002/evl3.256)
Supplement: Supplementary file 1 — Dataset S1: Levels of gene tree discordance across the concatenated, maximum likelihood suboscine phylogeny (1286 tips). Table S1: Species triads used in the focal analyses: 250‐sites species test (n = 130 triads). Table S2: Standardized regression coefficients as estimated by phylogenetic path analysis across subsampling schemes: 100‐sites subsampling (n = 179 triads), 250‐sites set (n = 130), and 500‐sites set (n = 63). Table S3: Standardized regression coefficients as estimated by phylogenetic path analysis across 100 bootstrap trees for the 250‐sites data set (n = 130 triads). Figure S1: Phylogenetic distribution of the triads originally selected for this study (shown in blue) and triads eventually retained in the primary data set (shown in orange) on the 1286‐species phylogeny of suboscine birds. Figure S2: The number of ABBA‐BABA informative sites sampled affects the likelihood of inferring a significant D‐statistic. Figure S3: Model for how different organismal and evolutionary factors influence levels of gene tree discordance. Figure S4: Three of the four models fit to our dataset using phylogenetic path analysis. Figure S5: The (A) number of D‐statistic informative SNPs and (B) and number of loci in which they fall across the 203 triads for which we could infer the D‐statistic. Figure S6: Correlations in D‐statistic estimates across different subsampling strategies. Figure S7: Results from model fitting in which introgression signal is treated as a binary variable (0 = no evidence for introgression, 1 = evidence for introgression). Figure S8: Descriptions of the triads originally selected for this study (n = 206; shown in blue) and triads eventually retained in the primary data set (n = 130; shown in orange): (A) Internode length of triad in millions of years (myr), (B) age of most recent common ancestor (MRCA) of triad, and (C) age of most recent common ancestor (MRCA) of triad plus the outgroup. Figure S9: Maps of the distributions of triads (A) with and [file EVL3-5-568-s001.docx]

# Supplementary Information

# Supplementary Methods

Our analytical approach uses the species tree of all suboscine birds in three primary analyses.

1. We compare gene trees to the species tree to determine levels of genealogical discordance.
2. We use the species tree to define species triads. For each of these species triads, we test for evidence of introgression.
3. We use the species tree to conduct a phylogenetic path analysis of predictors of genealogical discordance.

Uncertainty in our species tree can affect all three of these analyses. There are two ways to account for phylogenetic uncertainty. First, we can re-run our analyses using a tree inferred using a different approach. Second, we can re-run our analyses across either a bootstrap or posterior distribution of trees. As detailed below, we used both approaches to account for phylogenetic uncertainty across our three primary analyses.

In Harvey et al. (2020), the authors inferred a phylogeny for all suboscines using two methods. First, they concatenated all loci and inferred a species tree using ExaML. This is the species tree that we used in our current study. Harvey et al. (2020) then generated 100 bootstrap alignments from this concatenated alignment using RAxML and inferred a bootstrap topology for each alignment using ExaML. Using this bootstrap distribution, we both characterized how similar triads were across trees and we determined if our phylogenetic path analysis was robust to phylogenetic uncertainty. Running genealogical discordance analyses is computationally expensive, so we do not run it across this bootstrap distribution.

Second, Harvey et al. inferred gene trees for each locus and then inferred a species tree based on these gene trees using the coalescent-based method implemented in ASTRAL. This ASTRAL tree topology showed evidence for pathological errors, with many individuals being placed outside of their congeneric relationships. Subsequent analysis found that many of these errant individuals had high missing data. Removing individuals with high missing data resulted in a more sensible ASTRAL tree. However, this ASTRAL tree only represents 72% of suboscine diversity, whereas the ExaML tree represents 98.5% of suboscine diversity. The high levels of missing data in the ASTRAL tree prevents us from identifying true triads. After all, many of our focal species’ closest relatives are unsampled in this tree. In fact, of the 390 species in the 250-site dataset, 28% of them are missing in the ASTRAL tree. Given the limitations of the ASTRAL tree, we do not use it to define triads for introgression tests or for conducting phylogenetic path analyses. We do use it to determine if patterns of genealogical discordance are similar, irrespective of the approach for tree inference.

# Supplementary Results

Across nodes on the suboscine tree, between 7% and 100% (mean = 79%) of resolved gene trees were discordant with the consensus species tree inferred using ASTRAL (Fig. S12A). When mapped across space, the average gene tree discordance subtending the species occurring in each 200 km^2^ grid cell ranged between 26% and 99.5% (mean = 77%; Fig. S12B). These values are very similar to levels of gene discordance inferred using a concatenated species tree, suggesting that gene discordance is not a byproduct of errors in tree inference.

For each of the 100 bootstrap trees, we determined how many of the 206 triads in our original dataset were found across these bootstrap trees. Overall, we found high levels of similarity. Bootstrap trees contained an average of 97.9% of triads (range: 95.6% - 99.5%) found in the original dataset, and 189 of 206 triads were found in every bootstrap tree. In addition, bootstrap trees had an average of 3.7 unique triads not found in the consensus tree (range: 0 - 9 triads). Given the high similarity in triads recovered among the consensus tree and the bootstrap trees, we did not infer introgression across these unique triads. In our dataset of 130 triads with 250 ABBA-BABA informative sites, 119 triads were found in every single bootstrap tree. Forty-five of these 119 triads (37.8%) showed evidence for introgression. This prevalence of introgression is very similar to the patterns seen with all 130 triads; 38% of the 130 triads showed evidence for introgression.

For each of our 100 bootstrap trees, we determined if differences in triad composition and tree topology affected the results of our phylogenetic path analysis. Across the 100 trees, the “ILS + introgression” combined model was the best model for 86 trees (see Fig. S3 for models). Standardized regression coefficients and their significance levels across bootstraps compared to the consensus analysis (Table S3).

In summary, our analyses showed that accounting for phylogenetic uncertainty does not significantly change our quantitative or qualitative conclusions.

#

# Supplementary Datasets

# Dataset S1: Levels of gene tree discordance across the concatenated, maximum likelihood suboscine phylogeny (1286 tips). This phylogeny was inferred using ExaML. Levels of discordance were measured using phyparts on individual locus gene trees. Nodes with <80 Shimodaira-Hasegawa-like (SH-aLRT) support before calculating gene tree discordance. The first tree in the file has node labels indicating the number of supporting gene trees; the second tree has node labels indicating the number of conflicting gene trees.

# Dataset available at: https://github.com/singhal/bird_hyb/blob/main/phyparts.genetrees_80sh.T400F_AOS_HowardMoore.tre

# Supplementary Tables

**Table S1**: Species triads used in the focal analyses: 250-sites species test (*n* = 130 triads). Shown are species and sample names, number of ABBA-BABA informative sites prior to subsampling, D-statistic, Z-score, and p-value.

Table available at: <https://github.com/singhal/bird_hyb/blob/main/Table_S1.xlsx>

**Table S2:** Standardized regression coefficients as estimated by phylogenetic path analysis across subsampling schemes: 100-sites subsampling (*n* = 179 triads), 250-sites set (*n* = 130), and 500-sites set (*n* = 63). Our main text results focus on results from the 250-sites set. Bolded coefficients are significant. Patterns are fairly consistent across datasets, although the much smaller 500-sites dataset shows some departures from the other two datasets.

| Predictor | Predicted variable | 100-sites set | 250-sites set | 500-sites set |
| --- | --- | --- | --- | --- |
| geographic distance | introgression signal | **-0.126** | **-0.201** | -0.09 |
| climate velocity | introgression signal | **0.110** | **0.187** | -0.068 |
| latitude | introgression signal | -0.035 | **-0.115** | **0.256** |
| MRCA of hyb. pair | introgression signal | -0.024 | 0.027 | 0.014 |
| introgression signal | gene discordance | **0.152** | 0.040 | **0.105** |
| range size | gene discordance | -0.054 | 0.033 | **0.153** |
| internode length | gene discordance | **-0.664** | **-0.722** | **-0.774** |

**Table S3:** Standardized regression coefficients as estimated by phylogenetic path analysis across 100 bootstrap trees for the 250-sites data set (*n* = 130 triads). Shown are the minimum, maximum, and mean coefficients estimated for each correlation, as well as the percent of trees in which the coefficient was significant. Patterns are fairly consistent across bootstrap topologies, and mean coefficient estimates accord well with those found across the consensus species tree (Fig. 4A).

| Predictor | Predicted variable | Min. coef. | Max. coef. | Mean. coef. | % significant |
| --- | --- | --- | --- | --- | --- |
| geographic distance | introgression signal | -0.21 | -0.17 | -0.19 | 100% |
| climate velocity | introgression signal | 0.13 | 0.19 | 0.17 | 100% |
| latitude | introgression signal | -0.13 | -0.08 | -0.11 | 93% |
| MRCA of hyb. pair | introgression signal | 0.02 | 0.05 | 0.03 | 0% |
| introgression signal | gene discordance | 0.03 | 0.06 | 0.04 | 3% |
| range size | gene discordance | 0.03 | 0.05 | 0.03 | 100% |
| internode length | gene discordance | -0.74 | -0.72 | -0.72 | 0% |

#

# Supplementary Figures


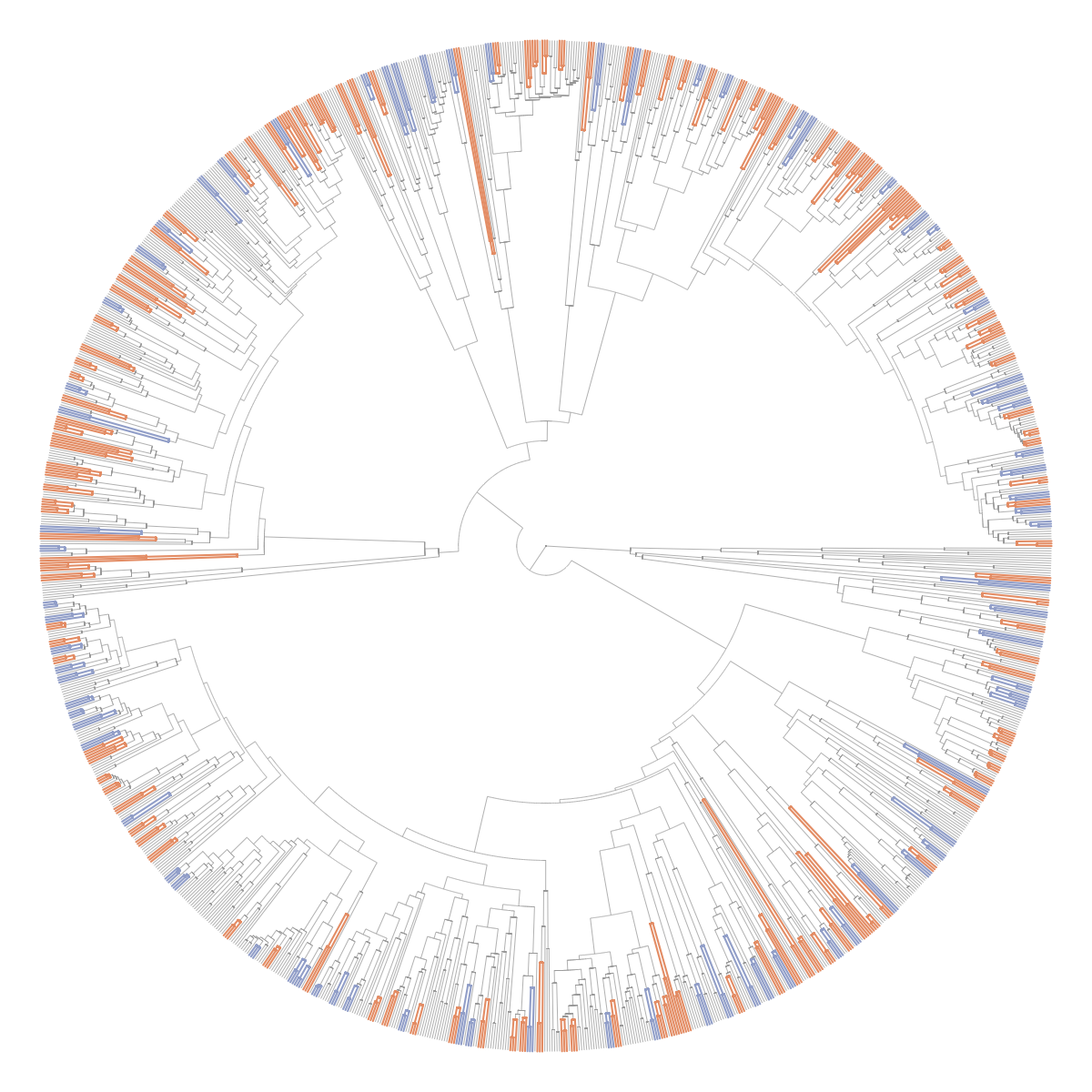


**Figure S1:** Phylogenetic distribution of the triads originally selected for this study (shown in blue) and triads eventually retained in the primary data set (shown in orange) on the 1286-species phylogeny of suboscine birds. The triads considered in this study span the full phylogenetic depth of suboscine birds.


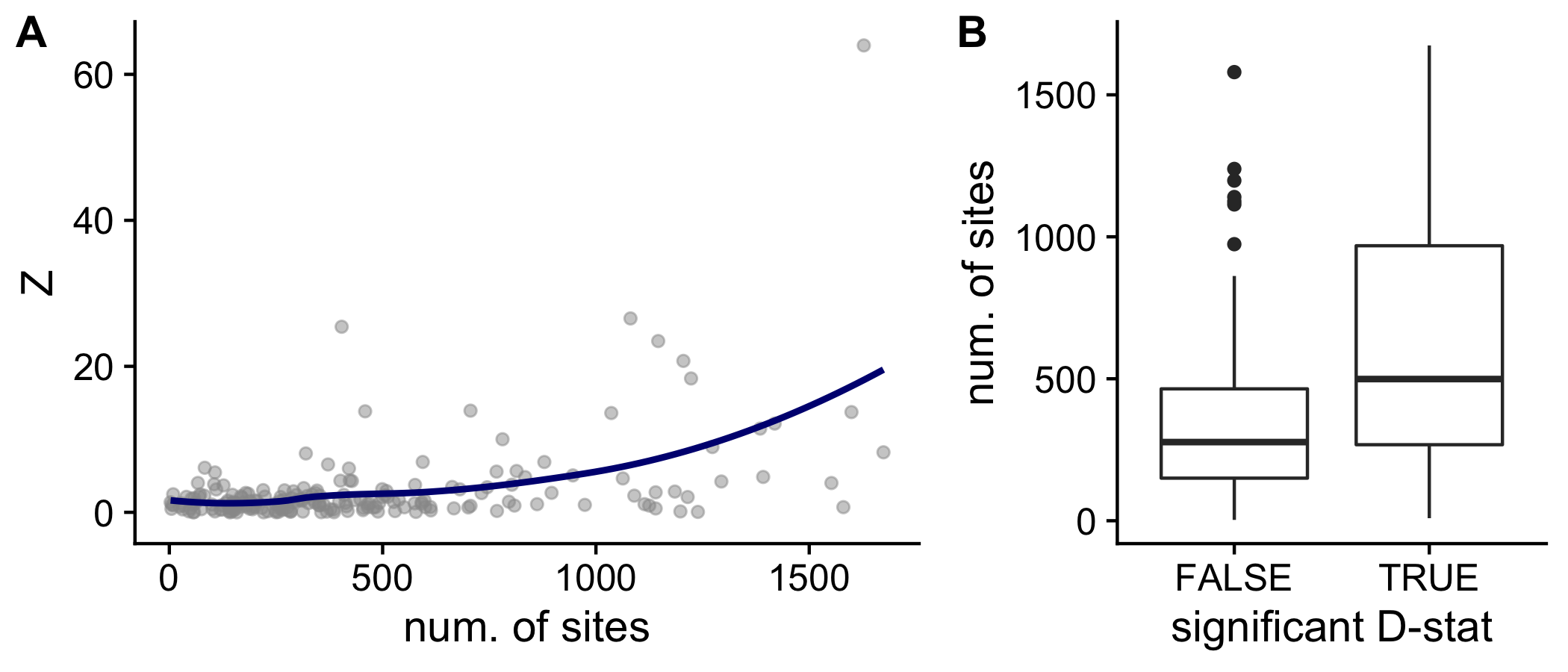


**Figure S2**: The number of ABBA-BABA informative sites sampled affects the likelihood of inferring a significant D-statistic. (A) More informative sites lead to higher Z-scores (Spearman’s rho = 0.33, p-val = 1.03e-6, n = 206). (B) Triads with a significant D-statistic were sampled at significantly more sites than those with non-significant statistics (ANOVA F_1,201_ = 24.64, p-val = 1.47e-6). Based on these results, we downsampled site datasets across triads to a shared value to ensure comparability across triads.


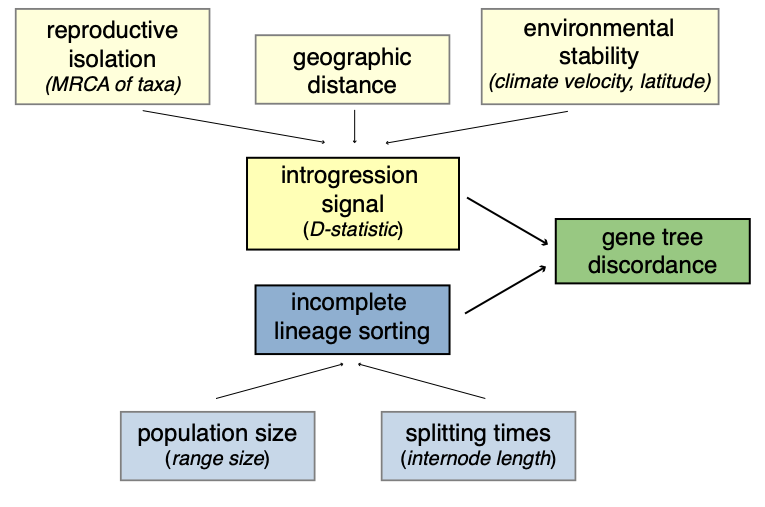


**Figure S3:** Model for how different organismal and evolutionary factors influence levels of gene tree discordance. Gene tree discordance is driven in part by incomplete lineage sorting and introgression. Shown in italics are proxies for these factors used in the current study.

**
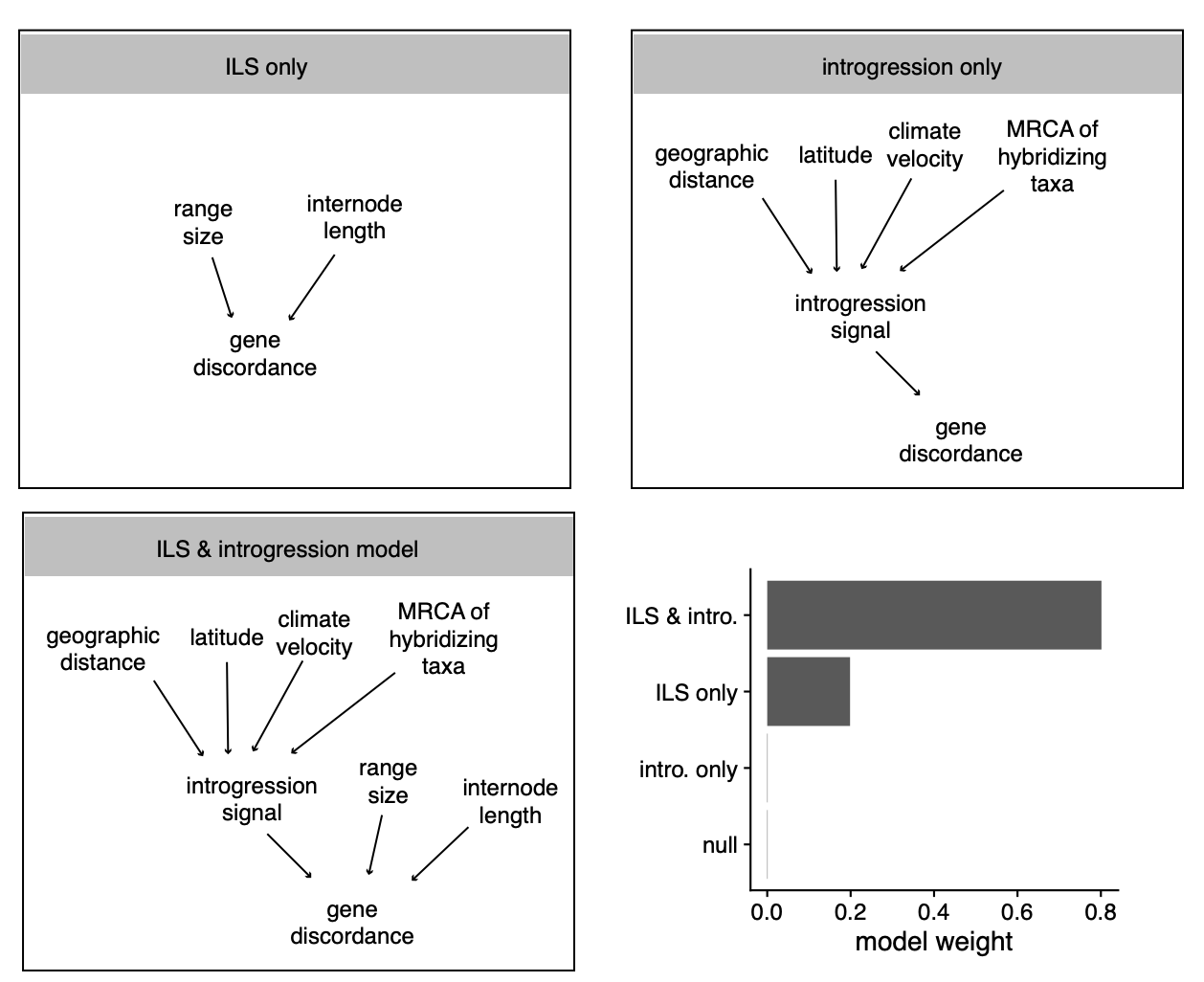
**

**Figure S4:** Three of the four models fit to our dataset using phylogenetic path analysis. The first model (ILS-only) models the contribution of ILS-related factors (internode distance and range size) to gene tree discordance. The second (introgression-only) models the contribution of introgression-related factors (divergence time between hybridizing pairs, absolute latitude, environmental instability, geographic distance between hybridizing pairs) on strength of introgression signal and thus gene tree discordance. The third model jointly models the contributions of ILS- and introgression-related factors. The fourth model (not shown) is a null model. Model choice gives the most weight to the most inclusive model that includes both ILS-related and introgression-related factors.


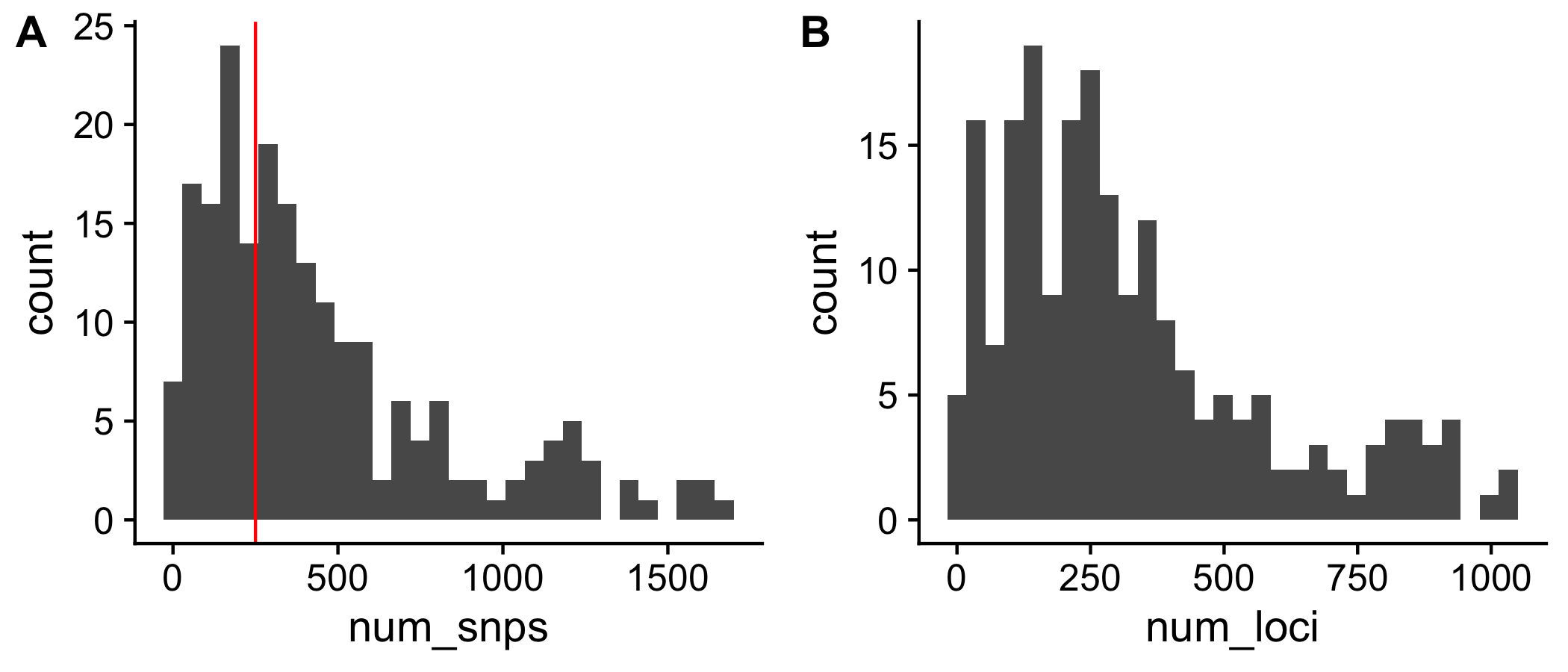


**Figure S5:** The (A) number of D-statistic informative SNPs and (B) and number of loci in which they fall across the 203 triads for which we could infer the D-statistic. In A, the red line marks 250 SNPs, the number of SNPs for which we subsampled data in our primary data set. Prior to filtering, triads had an average of 450.5 SNPs across 325 loci.


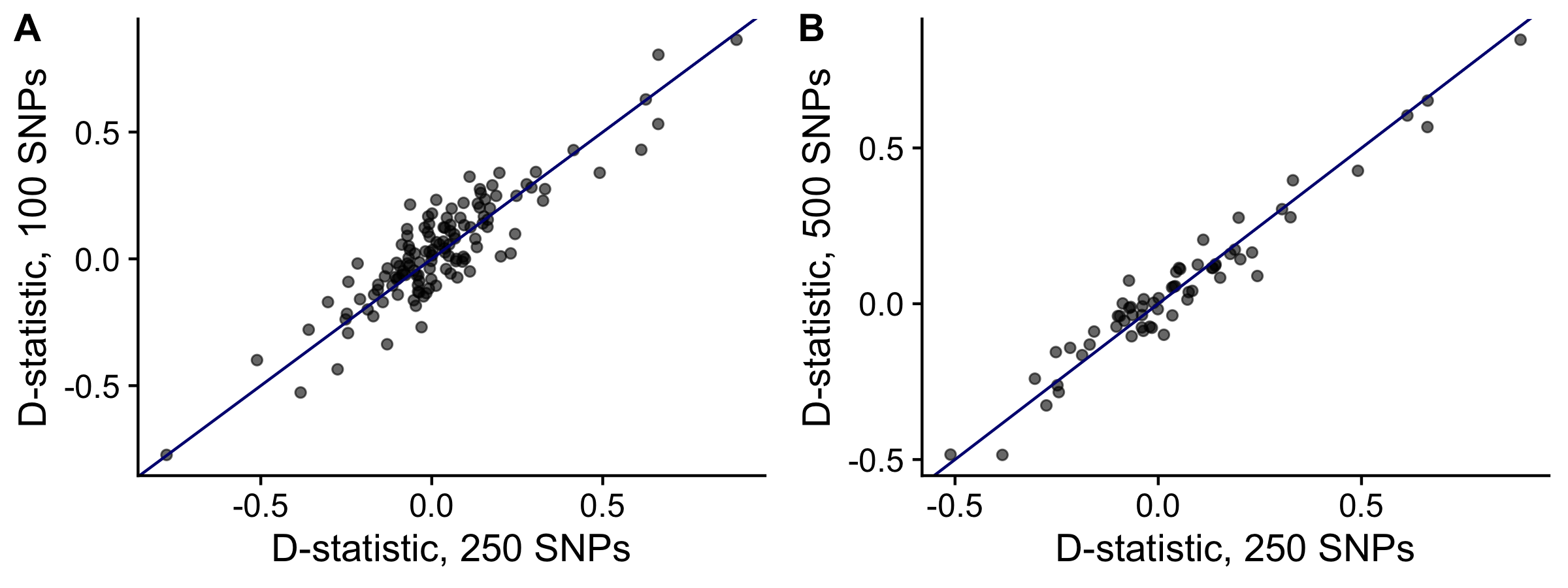


**Figure S6**: Correlations in D-statistic estimates across different subsampling strategies. (A) Subsampling for 250 versus 100 D-statistic informative SNPs (*r* = 0.89, *n* = 130) and (b) for 250 versus 500 SNPs (*r* = 0.97, *n* = 63). Subsampling does not appear to affect D-statistic estimates noticeably.


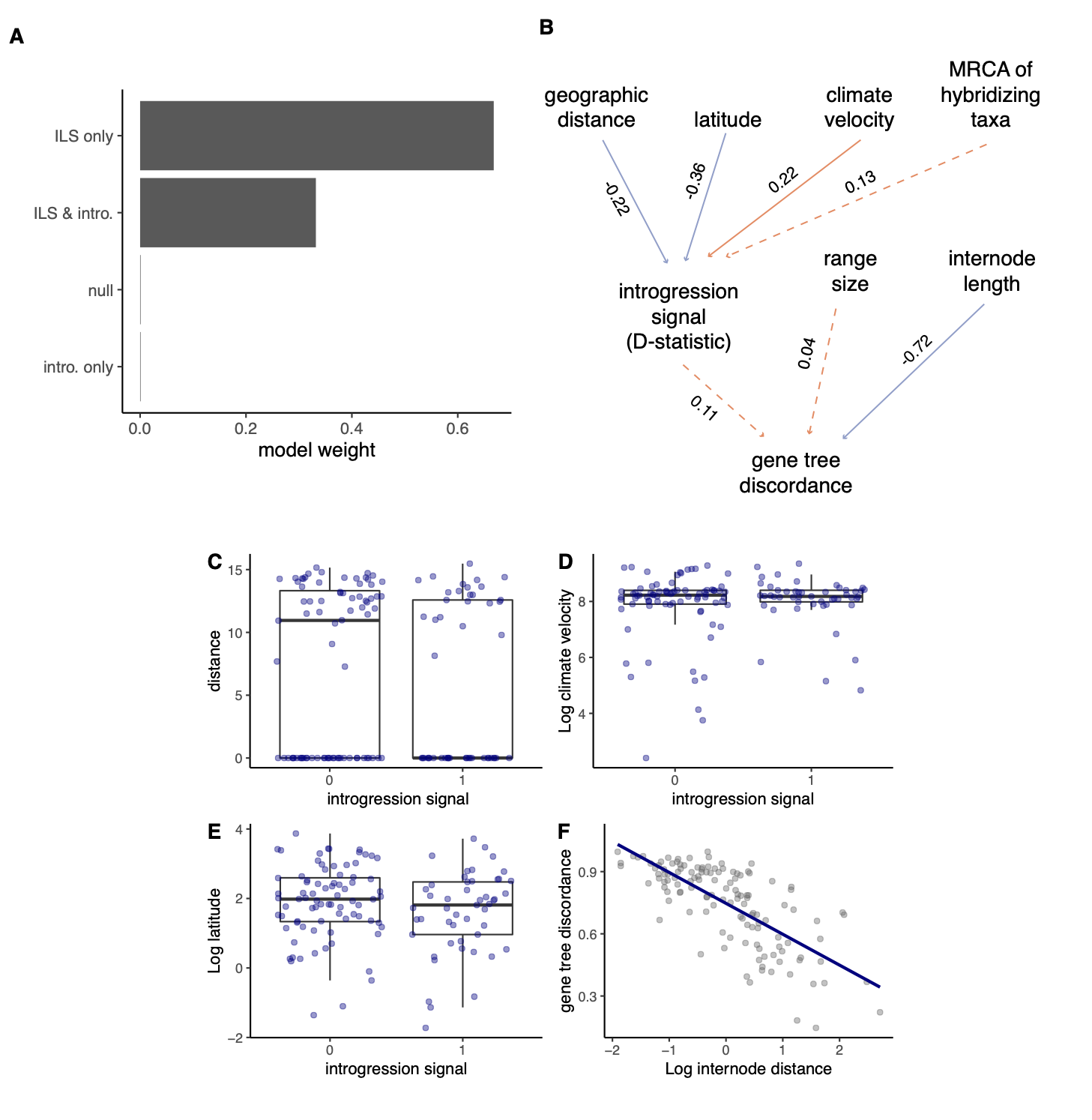


**Figure S7:** Results from model fitting in which introgression signal is treated as a binary variable (0 = no evidence for introgression, 1 = evidence for introgression). Here, evidence for introgression is determined if the Z-score for the D-statistic for introgression signal is significant at the α = 0.05 level. (A) Model weight is split between two models within 2 CICc of each other: the “ILS only” model and the “ILS + introgression” model. (B) The standardized regression coefficients for the average of the two best-fitting models. Orange and blue arrows indicate positive versus negative correlations, respectively; solid and dotted arrows indicate significant versus non-significant correlations, respectively. (C - F) The predictor variables against the predicted variables. Overall trends follow patterns found when treating introgression signal as a continuous variable (Fig. 4).


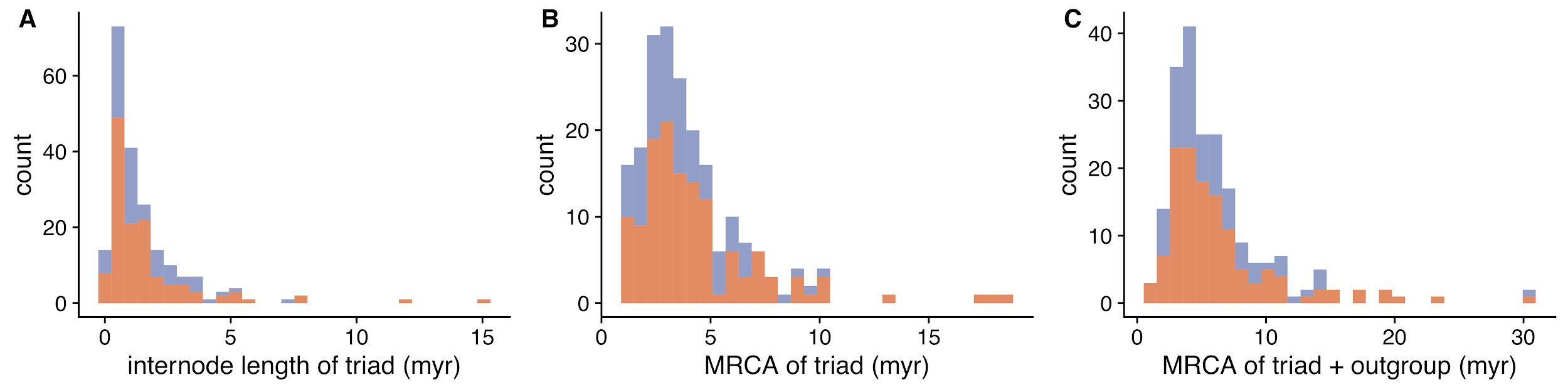


**Figure S8:** Descriptions of the triads originally selected for this study (*n* = 206; shown in blue) and triads eventually retained in the primary data set (*n* = 130; shown in orange): (A) Internode length of triad in millions of years (myr), (B) age of most recent common ancestor (MRCA) of triad, and (C) age of most recent common ancestor (MRCA) of triad plus the outgroup. These triads span a range of divergence histories but most represent relatively closely-related species (average MRCA of triad = 4.6 myr).

**
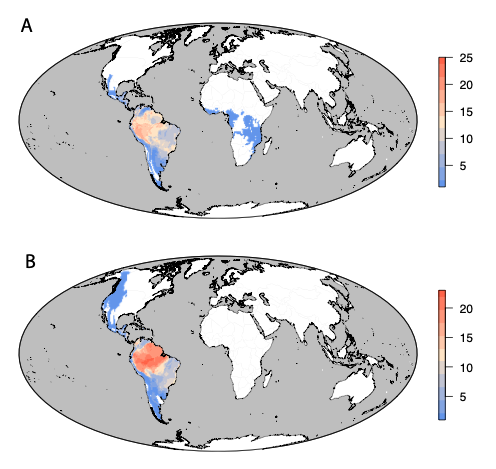
**

**Figure S9:** Maps of the distributions of triads **(A)** with and **(B)** without significant D-statistics. Each triad is mapped by combining polygons representing the distributions of only the two species with excess shared derived alleles.


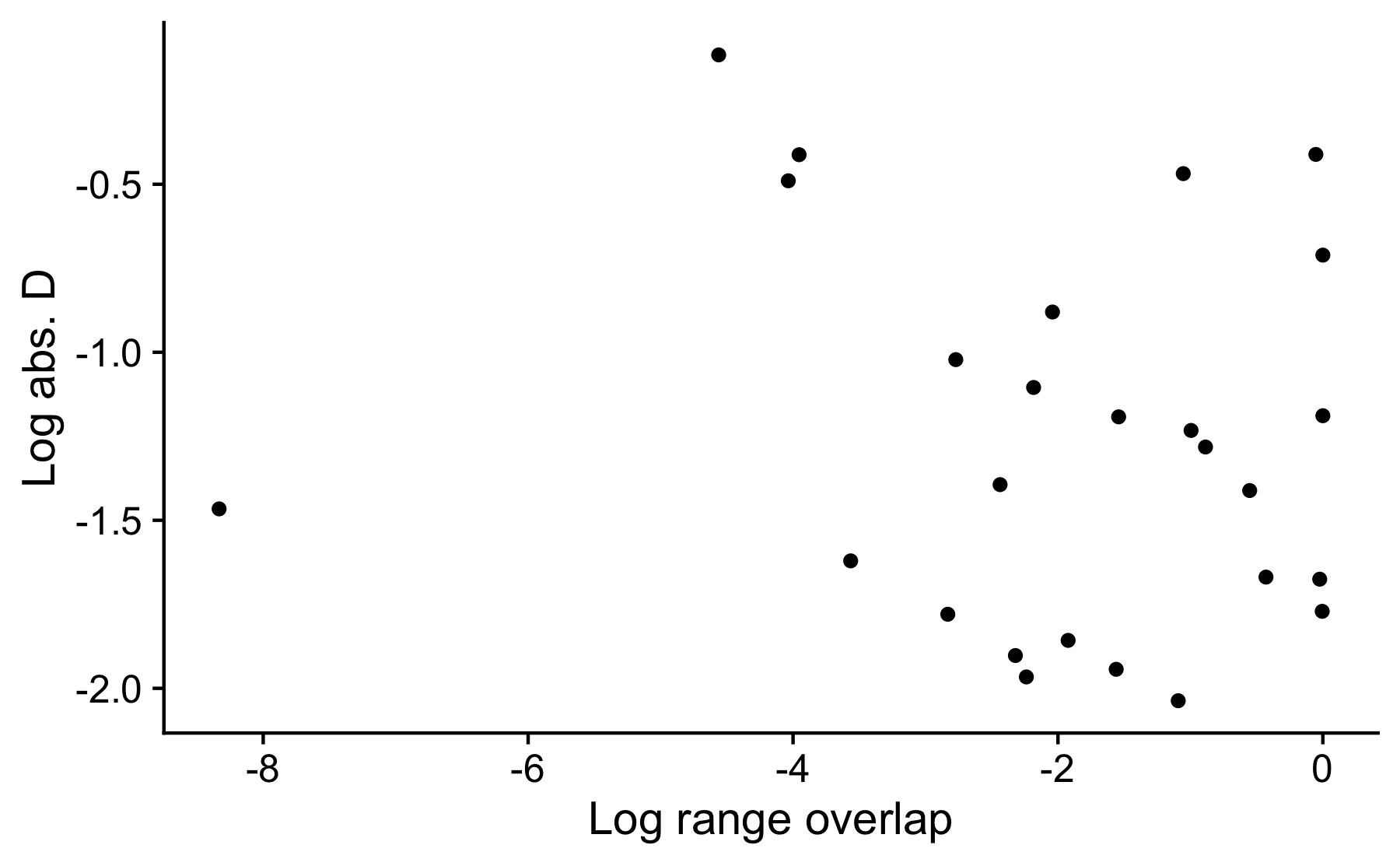


**Figure S10:** Correlation between log geographic range overlap and log absolute D-statistic of sympatric hybridizing pairs (n = 26). We find no evidence that hybridizing pairs with greater range overlap show more evidence for introgression (PGLS p = 0.6).


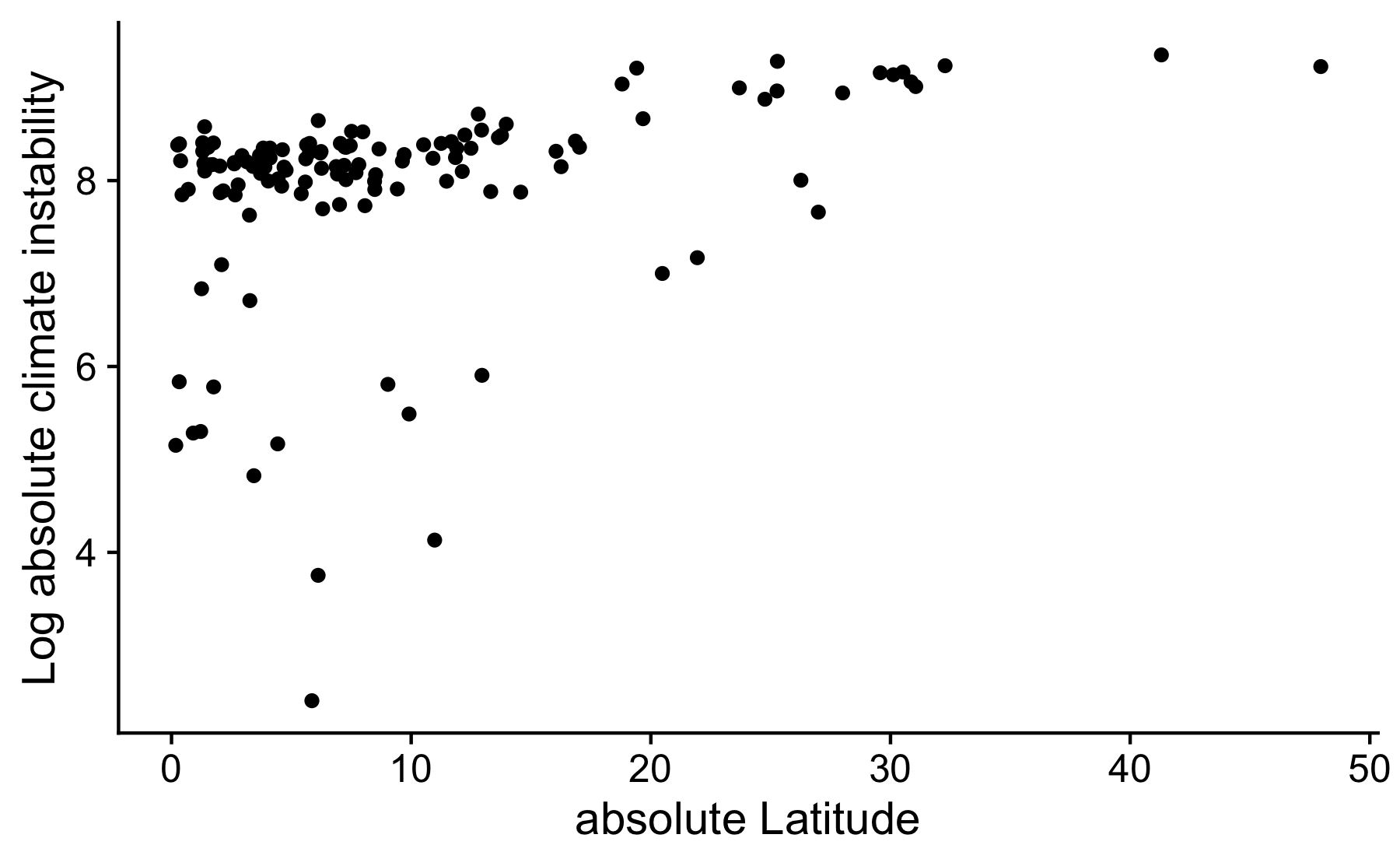


**Figure S11:** Correlation between absolute latitude and log absolute climate instability of triads in the 250 ABBA-BABA site dataset (n = 129). As expected, pairs at higher latitude show evidence for greater climate instability (*r* = 0.41, PGLS p-val = 0.005).

**
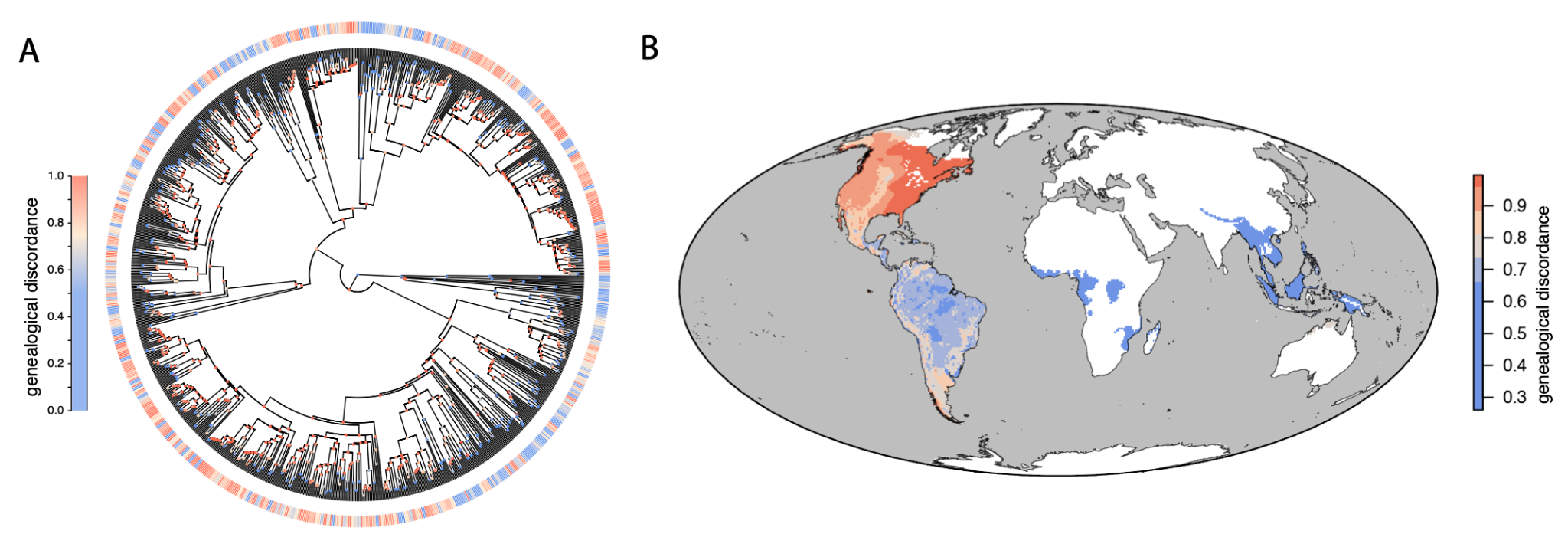
**

**Figure S12**: Variation in levels of gene tree discordance across a coalescent-based ASTRAL species tree. This is a 950-tip species tree, trimmed to remove individuals with high levels of missing data. **(A)** Gene tree discordance varies among nodes of the suboscine phylogeny and across species, based on a time-integrated summary of the history of gene tree discordance in their subtending lineages. Nodes are colored according to the proportion of discordant gene trees, whereas summary discordance values for species are depicted as colored bands in the encircling ring. Some nodes are colored white because there are no informative gene trees spanning that node. **(B)** The average level of gene tree discordance subtending the species occurring in a region varies geographically. The map is based on an equal-area projection with 200 km^2^ grid cells. Color scales differ between the two panels for ease of interpretation.
